# Geographic variation in wing size and shape of the grasshopper *Trilophidia annulata* (Orthoptera: Oedipodidae): morphological trait variations follow an ecogeographical rule

**DOI:** 10.1038/srep32680

**Published:** 2016-09-06

**Authors:** Yi Bai, Jia-Jia Dong, De-Long Guan, Juan-Ying Xie, Sheng-Quan Xu

**Affiliations:** 1 Institute of Zoology, Shaanxi Normal University, Xi’an, 710062, P.R. China; 2 School of Life Science, Taizhou University, Taizhou, 317000, P.R. China; 3 School of Computer Science, Shaanxi Normal University, Xi’an, 710062, P.R. China

## Abstract

A quantitative analysis of wing variation in grasshoppers can help us to understand how environmental heterogeneity affects the phenotypic patterns of insects. In this study, geometric morphometric methods were used to measure the differences in wing shape and size of *Trilophidia annulata* among 39 geographical populations in China, and a regression analysis was applied to identify the major environmental factors contributing to the observed morphological variations. The results showed that the size of the forewing and hindwing were significantly different among populations; the shape of the forewing among populations can be divided into geographical groups, however hindwing shape are geographical overlapped, and populations cannot be divided into geographical groups. Environmental PCA and thin-plate spline analysis suggested that smaller individuals with shorter and blunter-tip forewings were mainly distributed in the lower latitudes and mountainous areas, where they have higher temperatures and more precipitation. Correspondingly, the larger-bodied grasshoppers, those that have longer forewings with a longer radial sector, are distributed in contrary circumstances. We conclude that the size variations in body, forewing and hindwing of *T. annulata* apparently follow the Bergmann clines. The importance of climatic variables in influencing morphological variation among populations, forewing shape of *T. annulata* varies along an environmental gradient.

Environmental heterogeneity and ecological gradients can generate phenotypic variation in many organisms^1^,^2^,^3^. Understanding how environmental heterogeneity affects phenotypic patterns in organisms is a major focus in evolutionary ecology^4^,^5^,^6^,^7^. Under certain environment, phenotype changes can increase fitness in organisms^8^,^9^,^10^,^11^. Phenotypic clinal patterns associated with environmental gradients are often described as ecogeographical rules known as Bergmann’s rule or converse-Bergmann’ s rule^12^,^13^,^14^,^15^. Bergmann’s rule was initially used to explain the relationship between changes in the body size of endotherms and changes in latitude and altitude; it described a positive relationship between body size and latitude, in which smaller individuals are typically found at lower latitudes where climates are generally warmer. A number of studies have shown that the body size of insects along environmental gradients fit Bergmann clines or converse-Bergmann clines, but other studies have suggested that Bergmann’s rule might not work in insects^16^,^17^.

These ecogeographical rules have been extensively examined and convincingly demonstrated in insects^18^. However, morphological variations within insect species might reflect different patterns of dispersal and habitat availability coupled with different life-history types (e.g., hemimetabolism or holometamorphosis). The adaptive significance of these clines in insects has been fiercely debated^19^,^20^. Shelomi claimed that researches on these ecogeographical rules in insects should focus on widespread but contiguous populations to account for all sources of variation while minimizing errors^13^. Ideally, intraspecific morphometric analyses should sample from sufficiently large range to include obvious changes with both biotic and abiotic factors^21^,^22^. Numerous studies have focused on the factors influencing the size of adult insects, but little is known from the large geographical scale, specifically intraspecific variations in body size and shape of individuals that lives in diverse environments. It is necessary to examine the mechanisms that generate these patterns to understand how broad-scale geographic variations contribute to changes in body size^23^,^24^. Furthermore, studies are needed to examine whether patterns of insect size and clinal variations conform to Bergmann or converse-Bergmann clines and which environmental factors, if any, may be the key factors that determine shape variations^25^,^26^,^27^. Studies over large geographical ranges are expected to more precisely reflect the “true” clinal trends caused by ecological factors.

Grasshoppers are widely spread and environmental sensitive species whose body sizes and shapes change dramatically over a large geographical range^28^,^29^,^30^,^31^. Some studies have shown that grasshoppers are larger in cooler areas with longer growing seasons, whereas smaller body sizes are observed in warmer areas with shorter growing seasons^32^,^33^,^34^. These patterns of ecological variation are typical examples of Bergmann or converse-Bergmann clines. The debate on whether the size of grasshoppers along altitudinal or latitudinal gradients follows Bergman’s rule is ongoing^35^,^36^,^37^,^38^,^39^. However, researchers are more interested in the relationship between morphological variation and environmental factors over a large geographic area. The morphological characters frequently utilized in such studies include body mass, femur length, pronotum length, wing length and *etc.*^40^. Among these characters, wing size and shape are commonly used as indicators of the environmental changing and stress^41^,^42^.

The geophilous grasshopper *Trilophidia annulata* is wide geographically distributed across a steep-climatic gradient that ranges from the cold, temperate, northern region to the tropical climate south in China. These distributions provide an opportunity to study the influence of the environmental clines on intraspecific morphological variation. The genus *Trilophidia* includes five species, whose habitats include saturated grasslands, grassland savannas, irrigated areas and areas of sparse vegetation, and which is largely restricted to the Ethiopian and Oriental Regions^43^,^44^,^45^. Among these five species, *T. annulata* is the only widespread species distributed over the Oriental Region from West Pakistan to North Borneo, and the Palearctic Region of Mongolia, China, Korea and Japan. In this study, we explored the variations in wing size and shape in *T. annulata* over a large geographical range. We considered three questions: (1) how do the size and shape of wings change in *T. annulata*; (2) does the morphological variation of *T. annulata* along an environmental gradient meet certain ecogeographical rules; and (3) which environmental factors may contribute to the variations in wings.

## Results

### The geographical variation of body size (body length) of *T. annulata*

**Figure 1:**
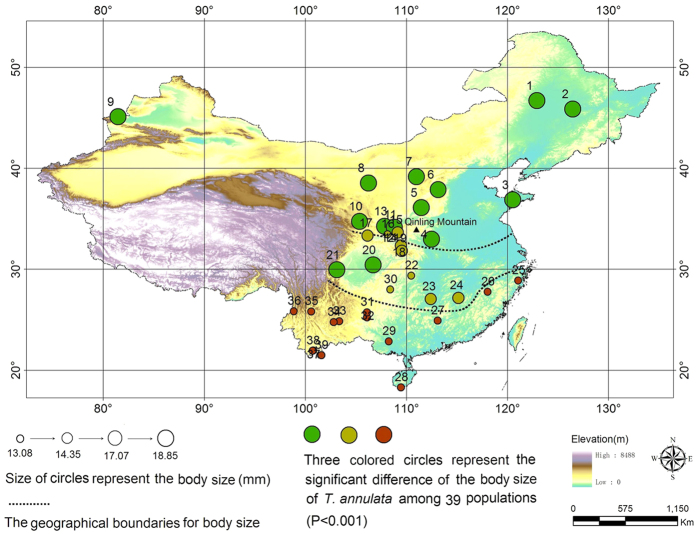
Sample sites and patterns of clinal variations in body size among the populations of *T. annulata*. The map in the figure was created by Yi Bai using ArcGIS software. URL: http://www.esri.com/software/arcgis/arcgisonline. *Scientific Reports* remains neutral with regard to jurisdictional claims in published maps.

The body size of male *T. annulata* varies from 13.08 mm to 18.85 mm in length within 39 populations. The one-way ANOVA showed that there were significantly different (F_(38,367)_ = 64.571, P < 0.001), a Tukey’s post-hoc HSD test results suggested that the 39 populations can be divided into 3 groups with significant difference (P < 0.001) (Fig. 1). Those body size range from 13.08 mm to 14.35 mm were mainly distributed in the south of 30° N, where the body size range from 14.35 mm to 17.07 mm were mainly distributed within region between 30° N and the south of Qinling mountains, while the body size range from 17.07 mm to 18.35 mm were mainly distributed in the north of Qinling mountains and northern China.

**Figure 2:**
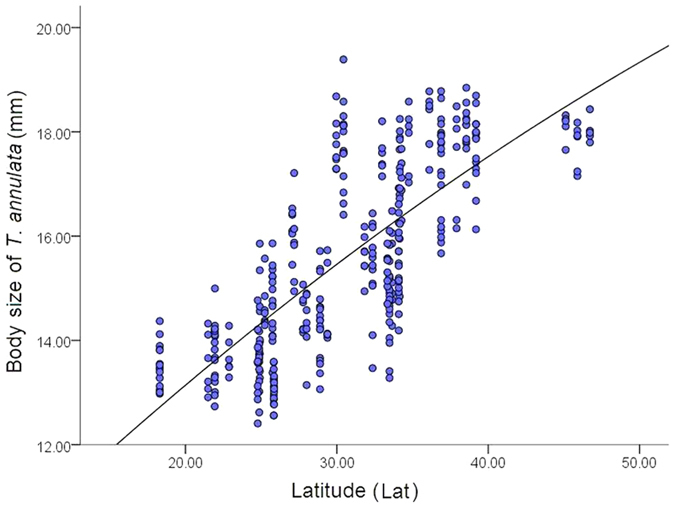
Relationships between body size and Latitude (Lat).

A regression analysis between body size of *T. annulata* and latitude showed that body size of male *T. annulata* are significant positive correlated with latitude (Lat) (r^2^ = 0.579, t = 22.42, P < 0.001) (Fig. 2), in precise, the body size of *T. annulata* increase along latitude. However, when latitude is higher than 40° N, the body size of *T. annulata* fell below expectations.

### Relationship between wing size and body size

**Figure 3:**
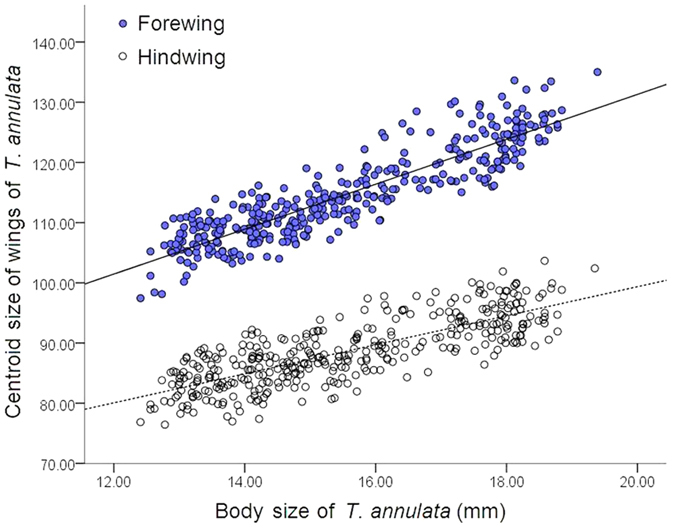
Relationships between body size and the centroid size (CS) of the forewing and hindwing.

The wing size was calculated on the basis of the centroid size. The regression analysis identified the relations between body size and wings size of male *T. annulata*, the results showed that body size were significant positively correlated with wings size (Forewing: r^2^ = 0.800, t = 38.303, P < 0.001; Hindwing: r^2^ = 0.625, t = 24.717, P < 0.001) (Fig. 3), that is, larger individuals with bigger wings. Wing size could be a proxy for body size in subsequent analysis.

### Wing shape variation

**Figure 4:**
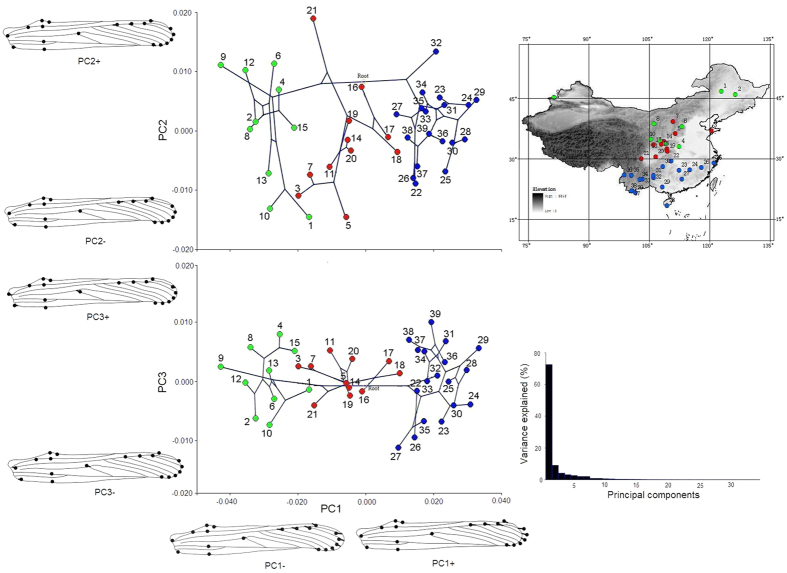
Pattern of clinal variations in forewing shape. The numbers in the picture identify the populations. The different colors indicate that the 39 populations identified 3 groups based on a cluster analysis of PC1 from the forewing shape data. Thin-plate spline analysis results are shown by the wing profile, which represents the deformations in wing shape in extreme conditions for each PC. The upper right corner of the figure shows 3 groups distributed on a map of China based on a cluster analysis. The lower right corner of the figure shows the variance explained by each PC. The map in the figure was created by Yi Bai using ArcGIS software. URL: http://www.esri.com/software/arcgis/arcgisonline. *Scientific Reports* remains neutral with regard to jurisdictional claims in published maps.

**Figure 5:**
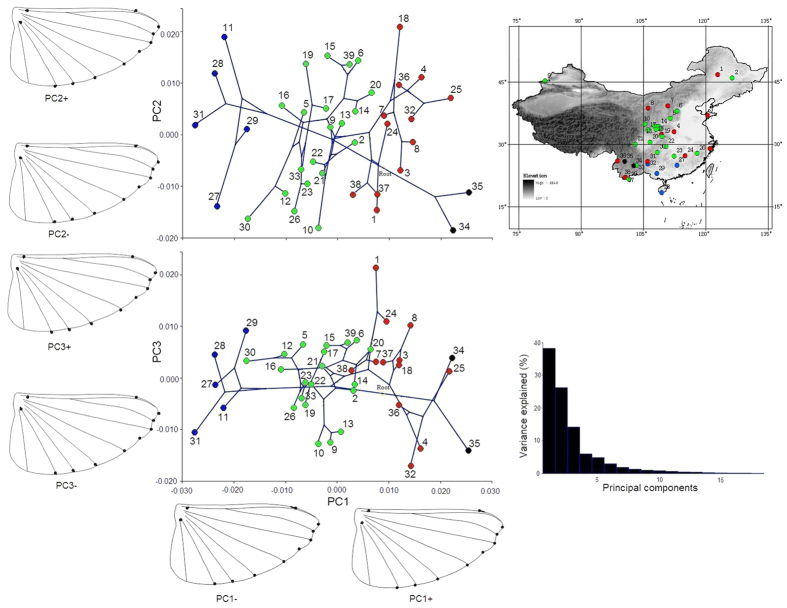
Pattern of clinal variations in hindwing shape. The numbers in the picture identify the populations, the different colors indicate that the 39 populations identified 4 groups based on a cluster analysis of PC1 from the hindwing shape data. Thin-plate spline analysis results are shown by the wing profile, which represents the deformations in wing shape in extreme conditions for each PC. The upper right corner of the figure shows 4 groups distributed on a map of China based on a cluster analysis. The lower right corner of the figure shows the variance explained by each PC. The map in the figure was created by Yi Bai using ArcGIS software. URL: http://www.esri.com/software/arcgis/arcgisonline. *Scientific Reports* remains neutral with regard to jurisdictional claims in published maps.

The wing shape data were analyzed via PCA and thin-plate spline analysis to find out the shape variation (Figs 4 and 5). The first three PCs account for 72.54%, 9.053%, and 4.16% of the variation, the cumulative variation explains 85.76% of the total shape variance of forewing. The PCA of shape variability from PC1 score showed that the forewing shape differences were highly significant among the 39 populations (F_2, 39_ = 146.562, P < 0.001); however, the shape variability from PC2 score (F_2, 39_ = 0.151, P > 0.05) and PC3 score (F_2, 39_ = 0.010, P > 0.05) were not significant. The 39 populations were clustered into three groups based on forewing shape (Fig. 4). The southern populations, which scattered along the positive PC1 axis (PC1+), were distributed in the south of 30° N, whereas populations north of the Qinling Mountains were mainly scattered on the negative PC1 axis (PC1−), and populations between 30 °N and the Qinling Mountains were scattered near the center of the PC1 axis. The thin-plate spline analysis shows that forewing shape deformation is mainly due to changes at the wing-end, and is based on the interaction between the Radius (Rs) and the edge of the wing (landmarks 3, 4, 5, 6, 7, 8, and 9) and on the branch of the Radius (landmarks 17, 18, and 19). Populations scattered on the side of the positive axis (PC1+) have shorter forewings with blunt-tip (shorter radial sector and smaller radial area), whereas populations scattered on the side of the negative axis (PC1−) have longer forewings with slightly projecting-tip (longer radial sector and bigger radial area). Populations scattered on the positive PC2 axis (PC2+) have broader-end and larger-medial-area forewings, whereas populations on the negative PC2 axis (PC2−) have narrower ends and smaller medial area. The forewing characteristics did not show significant change on the PC3 axis.

The first three PCs account for 38.23%, 26.25%, and 14.21% of the variation, the cumulative variation explains 78.69% of the total shape variance of hindwing. The PCA of shape variability from PC1 score showed that the hindwing shape variations are significant among the populations (F_3, 39_ = 67.202, P < 0.001); however, the shape variability from PC2 score (F_3, 39_ = 1.555, P > 0.05) and PC3 score (F_3, 39_ = 0.475, P > 0.05) were not significant. A cluster analysis showed that these 39 populations can be divided into four groups along the PC1 axis (Fig. 5). A thin-plate spline analysis showed that hindwing shape deformation was minimal along the PC1, PC2 and PC3 axes. The clustered groups based on hindwing shape are geographical overlapped, and populations cannot be divided into geographical groups.

### Characterization of the environmental niche

The PCA method was used to analyze 23 geographical and environmental factors associated with the 39 grasshopper populations. The first four PCs, cumulatively explaining 90.12% of the total variation, were used to illustrate the overall impact of environmental factors (Table 1). For PC1, the factors with relevant coefficients greater than 0.7 that showed a negative relationship were latitude (Lat), temperature seasonality (bio4) and annual temperature range (bio7). The factors with relevant coefficients greater than 0.7 and a positive correlation were annual mean temperature (bio1), minimum temperature of the coldest month (bio6), mean temperature of the driest quarter (bio9), mean temperature of the coldest quarter (bio11), annual precipitation (bio12), precipitation of the wettest month (bio13), precipitation of the wettest quarter (bio16) and precipitation of the warmest quarter (bio19), which were positively correlated with the PC1 scores. For PC2, the factors with relevant coefficients greater than 0.7 and a negative correlation were solar radiation (SR) and isothermality (bio3). The mean temperature of the wettest quarter (bio8) was positively correlated with PC3 scores.

### Relationship between clinal variations in wing size and environmental factors

A stepwise regression analysis was used to describe the relationships between the clinal variations in wing size and environmental factors (Table 2). The results showed that variations in the forewing size of *T. annulata* were significantly correlated with environmental factors (F_(2, 38)_ = 15.940, P < 0.001). Specifically, the forewing size was significantly correlated with PC1 and PC2 for environmental factors (forewing size vs. environmental PC1, (r^2^ = 0.470, t = −4.450, P < 0.001); forewing size vs. environmental PC2, (r^2^ = 0.470, t = 3.476, P = 0.001)). Meanwhile, hindwing size variations in *T. annulata* were also significantly correlated with environmental factors (F_(2, 38)_ = 16.333, P < 0.001). PC1 and PC2 showed that environmental factors and hindwing sizes were significantly correlated (hindwing size vs. environmental PC1, (r^2^ = 0.476, t = −4.443, P < 0.001); hindwing size vs. environmental PC2, (r^2^ = 0.476, t = 3.595, P = 0.001)) (Table 2).

**Figure 6:**
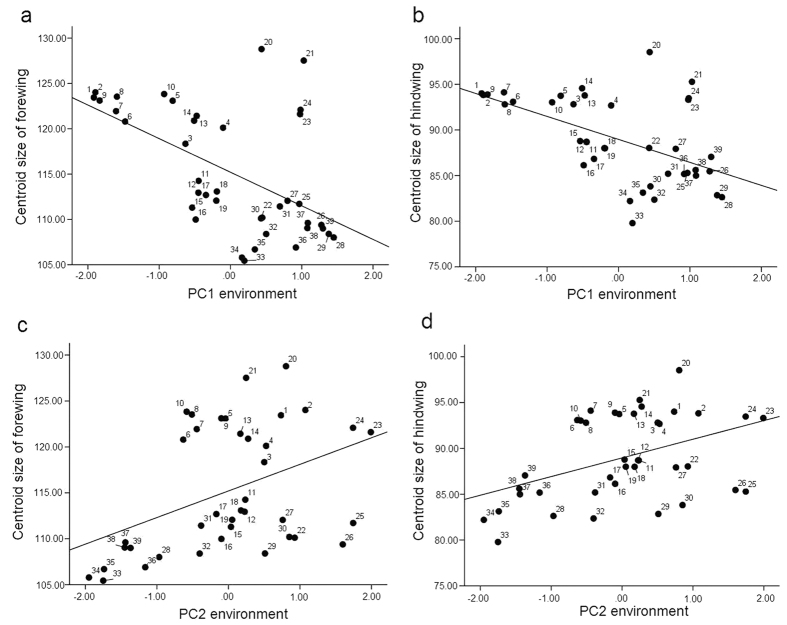
Relationship between the wing sizes of the 39 populations and the environmental PC scores. (**a**) Relationship between forewing centroid size (CS) and PC1 environmental scores. (**b**) Relationship between hindwing CS and PC1 environmental scores. (**c**) Relationship between forewing CS and PC2 environmental scores. (**d**) Relationship between hindwing CS and PC2 environmental scores.

The correlation between wing size and the environmental factor PCs was plotted with wing size on the vertical axis and the environmental factor PC scores on the horizontal axis (Fig. 6a–d). Figure 6(a,b) shows that the increased wings size of *T. annulata* corresponds to decreased PC1 scores for environmental factors (Forewing: r^2^ = 0.292, P < 0.001; Hindwing: r^2^ = 0.287, P < 0.001). As shown in Table 3, PC1 scores were negatively correlated with latitude, bio4 and bio7 but were positively correlated with bio1, bio6, bio9, bio11, bio12, bio13, bio16 and bio18, suggesting that the size of the forewings and hindwings increases with increasing latitude, bio4 and bio7; and that the size of the forewings and hindwings decreases with increases in bio1, bio6, bio9, bio11, bio12, bio13, bio16 and bio18.

Figure 6(c,d) shows that the wings size of *T. annulata* increased with increasing PC2 scores (Forewing: r^2^ = 0.178, P = 0.001; Hindwing: r^2^ = 0.188, P = 0.001), illustrating a positive correlation. As shown in Table 3, PC2 scores were negatively correlated with SR and bio3, suggesting that the size of the forewings and hindwings decreases with increases in SR and bio3.

### Relationships between wing shape and environmental factors

A stepwise regression was performed to analyze the relationships between wing shape and environmental factors (Table 3). PC1 scores for forewing shape were significantly correlated with environmental factors (F_(1, 38)_ = 75.356, P < 0.001). Specifically, there exist significant correlations between the PC1 score for forewing shape and PC1 score for environments factors (forewing shape PC1 vs. environmental PC1) (r^2^ = 0.671, t = 8.681, P < 0.001). The PC3 score for forewing shape was significantly correlated with environmental factors (F_(2, 38)_ = 10.186, P < 0.001). Specifically, there exist significant correlations between the PC3 score for forewing shape and PC2 and PC3 scores for environments factors (forewing shape PC3 vs. environmental PC2 (r^2^ = 0.361, t = −3.259, P = 0.002); forewing shape PC3 vs. environmental PC3 (r^2^ = 0.361, t = 3.123, P = 0.004)) (Table 3).

**Figure 7:**
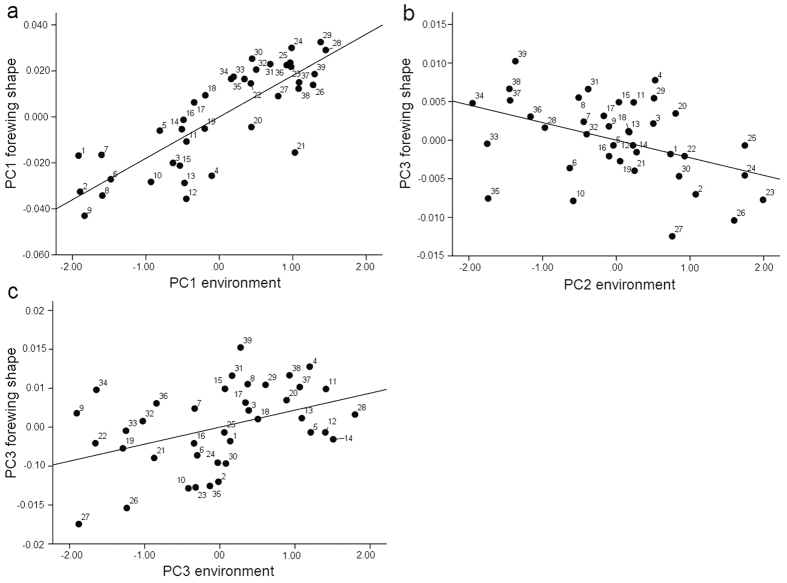
Relationship between the wing shapes of the 39 populations and environmental PC scores. (**a**) Relationship between PC1 forewing shape scores and PC1 environmental scores. (**b**) Relationship between PC3 forewing shape scores and PC2 environmental scores. (**c**) Relationship between PC3 forewing shape scores and PC3 environmental scores.

The correlations between the PCs of forewing shape and the environmental factors are shown in Fig. 7(a–c), with the forewing-shape data plotted on the vertical axes and the PC scores for environmental factors on the horizontal axes. Figure 7(a) shows that the PC1 scores for forewing shape and environmental factors are positively correlated (r^2^ = 0.671, P < 0.001). Figure 4 reveals that positive axes (PC1+) for forewing shape are characterized by shorter forewings with blunt-tip (shorter radial sector and smaller radial area), whereas shapes along the negative axis (PC1−) exhibited longer forewings with slightly projecting-tip (longer radial sector and bigger radial area). However, the PC1 scores for environmental factors were negatively correlated with latitude, bio4 and bio7 but were positively correlated with bio1, bio6, bio9, bio11, bio12, bio13, bio16, and bio18 (Table 1). The results suggest that *T. annulata* populations with shorter and blunt-tip forewings are mainly distributed in lower latitudes with higher temperatures and more precipitation, whereas lower seasonal temperature ranges and colder annual temperatures result in populations with longer forewings with slightly projecting-tip that are distributed at higher latitudes. Figure 7(b,c) shows that the PC3 scores for forewing shape were negatively correlated with the PC2 scores for environmental factors (r^2^ = 0.188, P = 0.002), but there was a positive correlation with the PC3 scores for environmental factors (r^2^ = 0.173, P = 0.004). The forewing shape changed on the PC2 and PC3 axes irregularly, and its relationship with environmental factors is therefore difficult to explain.

Table 3 shows that the first three PCs of hindwing shape in *T. annulata* were not significantly correlated with the PCs of environmental factors (P > 0.05). Thus, hindwing shape may not significantly change along the geographical and environmental gradients described in this study.

## Discussion

### Body size (wing size) changes significantly along environmental gradients

The larger body size of *T. annulata* with bigger wing size, so wing size could be used as a proxy for body size in this study, in line with other similar studies of insects^46^,^47^, and the variation in the body size of *T. annulata* is reflected in changes in wing size. Variability in body size is one of the most striking traits of most insects and strong relationships exist between body size and a variety of environmental factors associated with insects. Clinal variation in body size along latitudinal environmental gradients provides important insights into the adaptive challenges facing organisms and into their solutions for dealing with these challenges. This study shows that populations of *T. annulata* distributed from a cooler temperature zone to a tropical zone across several temperature gradients exhibit significant change in their body size and wing size in relation to different latitude and climate features. At lower latitudes with higher temperatures and more humidity conditions, *T. annulata* populations have smaller bodies and wings, whereas at higher latitudes with lower temperatures and drier conditions, they have larger bodies and wings.

These results can be described as Bergmann clines. However, such clines are not common in grasshoppers. Whitman^21^ reviewed the body sizes and geographic patterns of Orthoptera and stated that grasshopper body size varies spatially, both within and among species, with a tendency for warmer, drier areas, with longer growing seasons to contain relatively larger species^32^,^33^,^34^. Some grasshoppers follow Bergmann’s rule, with larger individuals or species existing at higher latitudes and altitudes, but most follow the converse-Bergmann’s rule, with larger individuals and species found at lower latitudes and altitudes. *T. annulata* is a species with a wide geographic distribution, and in a number of ways, it does not resemble many other grasshopper species that have limited or endemic distributions. *T. annulata* has the ability to move rapidly and it can effectively avoid predators. Members of this species are expected to be better adapted to climate changes^43^. Their distribution across different climate zones shows that the change of their morphological traits (body and wing size) and life history traits (growth, development and reproduction) can increase their fitness. Hassall^48^ suggests that some widely distributed and rapidly expanding insect species can quickly adapt to local climactic conditions. Their life history traits could alter accordingly, involving changes in morphology, physiology and/or behavior to improve their survival and reproductive success in a particular environment. Clinal variations of morphological traits, such as body and wing size along climate gradients, are more likely to follow Bergmann’s rule. *T. annulata* is a widely distributed species; the characteristics of its life cycle and behavior are similar to those of invasive species, its distribution model follows patterns of climate change, and its body size and wing size show clinal variations along climate gradients, this results support Bergmann’s rule.

Temperature is a major environmental factor explaining the distribution and individual development of insect. In the majority of cases, developmental temperature has the most significant influence on body size, and body size tends to be larger at lower temperatures. On the one hand, a larger body has a lower surface-to-volume ratio, so heat loss is minimized (or heat conservation is increased) when the temperature is lower. On the other hand, the metabolic rate of an organism is closely related to temperature: higher temperatures may speed up the metabolic rate, and to maintain a balanced energy budget, cell size must decline with increasing temperature. Assuming a constant number of cells, declining cell size should lead to declining body size^34^. Thus, *T. annulata* are smaller in high-temperature regions but are larger in low-temperature regions. Humidity (bio12, bio13, bio16 and bio18) is correlated with grasshopper development. Some studies have shown that more humid may be disadvantageous for the growth of grasshoppers. A moist environment during ovipositive season will shorten the post-diapause period of egg development of grasshoppers. In addition, humid conditions may exacerbate fungal pathogen (e.g., *Beauveria bassiana* Bals.) reproduction and dissemination, which could influence the growth of grasshoppers or even kill them^49^. However, direct evidence that humidity inhibits the growth of grasshoppers is lacking, so we did not extensively explore this aspect.

### Solar radiation affects the wing size of grasshoppers by affecting their development

Solar radiation is also an important factor that affects insect reproduction, growth and development. Some studies suggest that high-intensity solar radiation can cause mutations in reproductive cells, sperm abnormalities, increased DNA damage and oxidative stress^50^,^51^,^52^ and that it may have adverse effects on insect eggs and individual development and may even cause population numbers to drop^53^. Beasley^47^ demonstrated that solar radiation significantly affected the wings of grasshoppers such that wing size in late-maturing individuals decreased with increasing radiation levels. In this study, changes in *T. annulata* wing size and the PC2 scores for environmental factors were significantly and negatively correlated. PC2 environmental factors mainly consisted of solar radiation and isothermality. At lower latitudes, higher solar radiation and isothermality corresponded to smaller body and wing sizes; however, at higher latitudes, lower solar radiation and isothermality corresponded to larger body and wing sizes.

### Wing shape changes along environmental gradients

Large-scale spatial variation in the wing shapes of grasshoppers at the intraspecific level was considered in this study, which will help us discover the evolutionary patterns of wing shape under different environmental factors. Grasshopper wings change dramatically across various climates, and some of them have evolved long or short wings or even small wings that have resulted in a loss of flight^54^. In this study, the forewing shape of *T. annulata* significantly changed among the 39 populations, and wing shape deformation occurred mainly at the end of the forewing. Further integrating environmental characteristics of the population distribution areas, we found that individuals with longer forewings and slightly projecting-tip (longer radial sector and bigger radial area) were mainly distributed at higher latitudes and in flat areas where the climate was characterized by lower temperatures and drier conditions. On the contrary, individuals with shorter forewings and blunt-tip (shorter radial sector and smaller radial area) were mainly distributed in lower latitudes and mountainous areas characterized by higher temperatures and humid conditions. Microclimate and habitat characteristics are considered to be the main factors influencing grasshopper wing shape^40^,^55^. Mountainous environments may restrict the flying of grasshopper and lead to grasshoppers with smaller wings and weaker flight capabilities. Open areas may promote population intermixing and could favor grasshoppers with longer wings and better flight capabilities. Wing length is closely correlated with flight capability in insects^56^,^57^. Species that are good at flying tend to have narrower wings with longer ends. However, populations with shorter wings are often limited by geographic conditions and may depend more on jumping or walking as their main mode of locomotion. In this study, *T. annulata* populations distributed throughout various climate zones and terrains were examined. In the north, wide-open spaces and continuous habitats favor *T. annulata* with better flying capabilities, thus it has evolved larger and longer forewings with slightly projecting-tip in these regions. In the south, mountainous topography limit *T. annulata*’s ability to fly; thus, it has evolved smaller and shorter forewings with blunt-tip. The shape of the hindwing across these diverse environments changed no law to follows, perhaps its specific flight function has resulted in convergent evolution.

## Material and Methods

### Background

China is a vast land spanning many degrees of latitude, with complicated terrain and radical variations in climate. China also has a variety of temperature and rainfall zones. From north to south, climate types can be divided into five temperature zones: cold-temperate, mid-temperate, warm-temperate, subtropical and tropical zones. *T. annulata* belongs to the family Oedipodidae and occurs in all five temperature zones in China. Its habitats are found below an elevation of 2000 meters throughout grassland, open beach and farmland environments. *T. annulata* collected from different environments in China exhibit obvious differences in body length and wing size.

### Data collection

The specimens of *T. annulata* used in this study came from two sources. Fresh specimens were collected during autumn in 2010 to 2013 from all types of environments in China. These fresh specimens were preserved in 80% alcohol. The pinned specimens were from the museum of the Institute of Zoology, Shaanxi Normal University. Male adults of *T. annulata* were selected, all samples were arranged, classified and numbered according to collection times and location. We examined at least 5 individuals for each population, total in 368 males from 39 collection sites, which were furnished to the morphological analysis. The locality and specimen numbers of each collection site were listed in Table 4.

Specimens were softened for 8 hours in 10% potassium hydroxide, then the right forewing and hindwing were dissected, cleaned and flatten between 2 clean glass slides. Each specimen was given a unique code number. Images of the right forewing and hindwing of all 368 specimens were captured using a Sony DSC-H5 camera attached to a copy stand, with fixed focus, camera angle and magnification. Images were used in the subsequent morphological analysis.

Body size (body length, the length from tip of the head to the end of hind femur) was measured using handheld vernier callipers (accurate to 0.02 mm). This body length, including the length of head, dorsal pronotal and hind femur, were closely correlated with body size and other size metrics in grasshoppers, and it is more reliable than body length (from tip of the head to the end of genitalia), which may change while specimens dried^58^,^59^.

**Figure 8:**
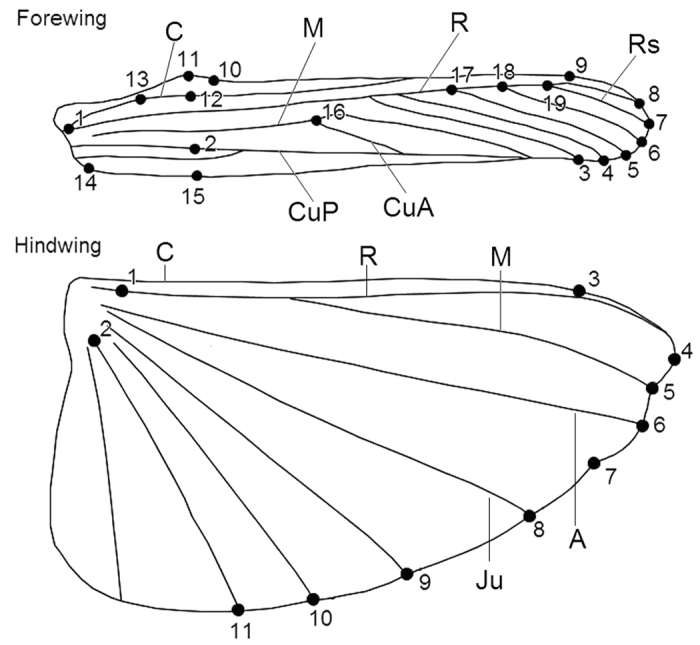
Wing venation of male *T. annulata* with major vein labelled and landmark distributions on the wings. C, costa vein; M, medial vein; R, radius vein; Rs, radial sector vein; CuA, cubitus anterior vein; CuP, cubitus posterior vein; A, anal vein; Ju, jugal vein.

The landmark-based geometric morphometrics method was applied to study the clinal change in wing size and shape. We set landmarks at the intersections of wing veins with the wing margin, intersections of cross veins with major veins and vein branch points, which is the same kind of landmarks used by Rohlf and Slice^60^. All the veins are homologous and regarded as classification characters of grasshopper^61^. A total of 19 landmarks on the forewing and 11 landmarks on the hindwing positioned at vein intersections or terminations (Fig. 8) were identified and digitized using TpsDig 2.10^62^. These landmarks were used to match the *x and y* coordinates in a Cartesian space^63^. Descriptions of the 19 landmarks on forewings and 11 landmarks on hindwings are provided in Table 5. Based on the landmarks, the wing size was calculated on the basis of the centroid size (CS; the square root of the sum of the squared distances between each landmark and the wing centroid). The CS of the wings was obtained for morphological analysis.

### Morphometric and Statistical Analyses

#### Body size and wing size anlysis

The regressive analysis method was used to study the relationships between the body size and the latitude. The differences in body length among these 39 populations were tested using one-way ANOVA (Tukey’s post-hoc HSD test). We also used ArcGIS software to draw a map of the *T. annulata* collection sites. The body size of each population was plotted on the map, and indicated using size and color of circles (Fig. 1). The relationship between the body size and latitude (LAT), centroid size (CS) of the wings was examined by a regressive analysis (Figs 2 and 3).

#### Wing shape analysis

To examine wing-shape variation, digitized landmark data were subjected to generalized Procrustes superimpositions to standardize the size of the landmark configurations and eliminate differences caused by translation and rotation^60^. Morphometric analyses were conducted using the IMP software package^62^. A single reference shape configuration for each population (i.e., a consensus wing) was obtained. The consensus wing was used for aligning all individual shape configurations and for computing the shape components (i.e., partial warps and the uniform component). We used a consensus shape configuration for each population to build a matrix for the 39 populations. The resulting weight matrix^64^ was then used to explore the shape changes according to the means of a multivariate principal component analysis (PCA). The PCA was conducted using MorphoJ1.06d software^65^. PCA is a method to reduce a large set of variables to a few dimensions that represent most of the variation in the data. Principal component scores are the projections of the shapes onto the low-dimensional space spanned by the eigenvectors. The major axis of variation can be plotted as two- or three-dimensional graphics and allow for the assessment of group differences^55^,^66^. To test for wing shape differences among populations, we then performed multivariate analysis of variance (MANOVA) on scores from all PC axes, with group identity as a fixed factor. This MANOVA was implemented using SPSS 13.0. Major shape changes in the projected lateral views were illustrated using a thin-plate spline analysis^67^. The visual representation of the shape differences described by the principal component axis was produced by regressing the shapes (the weighted matrix of the partial warp scores) onto the specimen scores on the first three principal component axes. This representation permitted the splines of the shape change to be associated with the positive and negative values of a vector component.

#### Cluster analysis on the shape data to establish the relationship among populations

Principal component scores (the forewing and hindwing extracts from the first three PCs explained 85.75% and 78.68%, respectively, of the total variance) from the PCA were exported using the “export dataset” options of MorphoJ. The PC scores were then imported into Mesquite software for a cluster analysis. The cluster analysis was based on the average of several distances between each population, with PC scores used to establish a matrix. Then, the relationships among the populations were further summarized based on the unweighted pair-group method with arithmetic averages (UPGMA). Finally, the cluster results were imported into MorphoJ, and using the “Map onto Phylogeny” option. The PCA and cluster results for the forewing and hindwing are displayed in Figs 4 and 5.

#### Relationship between environmental niches and morphological traits

The geographical coordinates of the 39 populations were imported into DIVA-GIS7.5 software to extract the environmental factors for each site^68^. Environmental data sources were retrieved from the WorldClim Global Climate database at a 30-second resolution^69^. This data source, with 20 environmental factors, contains annual trends, annual seasonal trends, seasonality and extreme or limiting environmental factors, and includes elevation (m), annual mean temperature (all temperatures in °C), mean diurnal range, isothermality, temperature seasonality, maximum temperature of the warmest month, minimum temperature of the coldest month, annual temperature range, mean temperature of the wettest quarter, mean temperature of the driest quarter, mean temperature of the warmest quarter, mean temperature of the coldest quarter, annual precipitation (all precipitation levels in mm), precipitation of the wettest month, precipitation of the driest month, seasonal precipitation, precipitation of the wettest quarter, precipitation of the driest quarter, precipitation of the warmest quarter and precipitation of the coldest quarter. Several studies have suggested that latitude, longitude and solar radiation may significantly influence the morphology of insects^70^,^71^. In this study, we chose the 20 environmental factors described above and added data for latitude, longitude and annual solar radiation. These 23 geographical environmental factors were considered when assessing the differences observed in the morphological traits of the grasshoppers. The descriptive functions of SPSS 13.0 were employed to log_10_-transform all environmental data to eliminate the dimensional differences among the data^72^. Then, we performed a principal components analysis (PCA; SPSS 13.0) on the environmental data to characterize the environmental niche (i.e., the environmental space) occupied by each population^73^. We calculated the mean scores of all principal components (PCs) in each population and used these mean scores to conduct subsequent analyses (Table 1).

The forewing and hindwing scores for the first three PCs in the 39 populations of *T. annulata* sampled were used along with the first four PCs of the environmental factors to develop a stepwise regression model. The details are as follows: the first three forewing and hindwing PC scores represent the major morphological differences among the 39 populations. The first four PC scores of the environmental factors represented the major environmental differences among the 39 sites (populations). Then, a stepwise regression was used to detect the morphological clinal rules describing the relationships between the forewings and hindwings of *T. annulata* and the environmental factors (Tables 2 and 3).

## Additional Information

**How to cite this article**: Bai, Y. *et al*. Geographic variation in wing size and shape of the grasshopper *Trilophidia annulata* (Orthoptera: Oedipodidae): morphological trait variations follow an ecogeographical rule. *Sci. Rep.* **6**, 32680; doi: 10.1038/srep32680 (2016).

## Figures and Tables

**Table 1:** Eigenvalues, percentage of total variance explained, and principal component loadings from analysis of environmental data extracted from the localities of the 39 populations.

Variable code	Variable type	PCA axes			
		1	2	3	4
	Eigenvalue	12.114	4.444	2.567	1.602
	% total variance explained	52.668	19.320	11.161	6.966
Lat	Latitude	**−0.931**	0.292	−0.009	0.064
Long	Longitude	−0.118	0.606	0.178	0.577
SR	Solar radiation	−0.180	**−0.764**	0.221	0.150
Alt	Elevation of site	−0.186	**−**0.684	−0.587	−0.215
Bio 1	Annual mean temperature	**0.940**	−0.201	0.259	−0.071
Bio 2	Mean monthly temperature range	−0.594	−0.499	0.075	0.028
Bio 3	Isothermality: (Bio2/Bio7) *100	0.459	**−0.821**	0.048	0.032
Bio 4	Temperature seasonality (STD *100)	**−0.842**	0.520	0.028	0.081
Bio 5	Maximum temperature of warmest month	0.455	0.425	0.678	−0.174
Bio 6	Minimum temperature of coldest month	**0.948**	−0.220	0.134	−0.103
Bio 7	Temperature annual range (Bio5–Bio6)	**−0.900**	0.357	0.045	0.062
Bio 8	Mean temperature of wettest quarter	0.610	0.026	**0.700**	0.121
Bio 9	Mean temperature of driest quarter	**0.948**	−0.260	0.120	−0.077
Bio 10	Mean temperature of warmest quarter	0.692	0.383	0.591	−0.058
Bio 11	Mean temperature of coldest quarter	**0.926**	−0.328	0.147	−0.084
Bio 12	Annual precipitation	**0.923**	0.180	−0.277	0.143
Bio 13	Precipitation of wettest month	**0.798**	0.025	−0.253	0.489
Bio 14	Precipitation of driest month	0.679	0.540	−0.356	−0.105
Bio 15	Precipitation seasonality (coefficient of variation)	−0.496	−0.372	0.183	0.723
Bio 16	Precipitation of wettest quarter	**0.873**	0.008	−0.264	0.360
Bio 17	Precipitation of driest quarter	0.687	0.525	−0.343	−0.112
Bio 18	Precipitation of warmest quarter	**0.788**	−0.082	−0.308	0.408
Bio 19	Precipitation of coldest quarter	0.659	0.532	−0.357	−0.075

**Table 2:** Results of the stepwise linear regressions predicting wing size changes in *T. annulata* based on a variety of environmental measures.

	Coefficient	T	P
**(a) Dependent with Forewing size, Model: R^2^ = 0.470; F = 15.940; df = 2, 38, P < 0.001**			
Intercept	115.206	140.172	0.000
PC1 environment	−3.705	−4.450	0.000
PC2 environment	2.894	3.476	0.001
PC3 environment	—	—	—
PC4 environment	—	—	—
**(b) Dependent with Hindwing size, Model: R**^**2**^** = 0.476; F = 16.333; df = 2, 38, P < 0.001**			
Intercept	88.963	159.029	0.000
PC1 environment	−2.518	−4.443	0.000
PC2 environment	2.038	3.595	0.001
PC3 environment	—	—	—
PC4 environment	—	—	—

(a) Dependent variable: forewing size, independent variables: PC1, PC2, PC3, PC4 for environmental measures; (b) dependent variable: hindwing size, independent variables: PC1, PC2, PC3, PC4 for environmental measures.

**Table 3:** Results of the stepwise linear regressions predicting wing shape changes in *T. annulata* based on a variety of environmental measures.

(a) Dependent with PCs Forewing shape			
	Coefficient	T	P
Intercept	−5.128^−11^	0.000	1.000
PC1 environment	0.018	8.681	0.000
PC2 environment	—	—	—
PC3 environment	—	—	—
PC4 environment	—	—	—
PC2 Forewing shape, Model: no factors entry into model.			
**PC3 Forewing shape, Model: R**^**2**^** = 0.361; F = 10.186; df = 2, 38; P < 0.001**			
Intercept	−1.613^−8^	0.000	1.000
PC1 environment	—	—	—
PC2 environment	−0.002	−3.259	0.002
PC3 environment	0.002	3.123	0.004
PC4 environment	—	—	—
**(b) Dependent with PCs Hindwing shape**			
PC1 Hindwing shape, Model: no factors entry into model.			
PC2 Hindwing shape, Model: no factors entry into model.			
PC3 Hindwing shape, Model: no factors entry into model.			

(a) Dependent variables: PC1, PC2, PC3 for forewing shape, independent variables: PC1, PC2, PC3, PC4 for environmental measures; (b) dependent variables: PC1, PC2, PC3 for hindwing shape, independent variables: PC1, PC2, PC3, PC4 for environmental measures.

**Table 4:** Populations identifiers (ID), locality and specimen numbers (N) of each collection site for *T. annulata* complex specimens analyzed in this study.

Populations ID	Locality	Province	Coordinates	N
1	Jalaid Banner	Nei Monggol	46°43′9″N, 122°55′9″E	5
2	Harbin	Heilongjiang	45°53′18″N, 126°27′26″E	6
3	Penglai	Shandong	36°53′34″N, 120°31′24″E	20
4	Nanyang	Henan	32°59′7″N, 112°28′22″E	6
5	Linfen	Shanxi	36°6′31″N, 111°28′13″E	6
6	Shouyang	Shanxi	37°54′30″N, 113°8′25 ″E	6
7	Fugu	Shaanxi	39°11′29″N, 110°58′17″E	15
8	Yinchuan	Ningxia	38°33′11″N, 106°14′30″E	12
9	Bortala	Xingjiang	45°7′17″N, 81°26′29″E	5
10	Gangu	Gansu	34°45′18″N, 105°21′52″E	6
11	Zhouzhi	Shaanxi	34°9′16″N, 108°13′36″E	5
12	Louguantai	Shaanxi	34°5′38″N, 108°18′25″E	18
13	Meixian	Shaanxi	34°16′24″N, 107°50′28″E	5
14	Chang'an	Shaanxi	34°9′33″N, 108°47′57″E	8
15	Zhashui	Shaanxi	33°39′10″N, 109°8′20″E	7
16	Foping	Shaanxi	33°28′41″N, 108°7′6 ″E	20
17	Lueyang	Shaanxi	33°20′26″N, 106°9′19″E	8
18	Pingli	Shaanxi	32°21′33″N, 109°26′23″E	12
19	Zhenping	Shaanxi	31°48′58″N, 109°31′48″E	7
20	Guang'an	Sichuan	30°26′38″N, 106°41′31″E	15
21	Ya'an	Sichuan	29°57′35″N, 103°6′53″E	8
22	Zhangjiajie	Hunan	29°22′32″N, 110°28′8″E	6
23	Hengyang	Hunan	27°3′21″N, 112°24′21″E	6
24	Jishui	Jiangxi	27°10′51″N, 115°8′7″E	6
25	Linhai	Zhejiang	28°53′32″N, 121°4′11″E	18
26	Wuyishan	Fujian	27°46′20″N, 118°1′46″E	6
27	Ruyuan	Guangdong	24°54′50″N, 113°4′58″E	9
28	Sanya	Hainan	18°18′5″N 109°27′9″E	16
29	Nanning	Guangxi	22°52′31″N 108°14′34″E	6
30	Yinjiang	Guizhou	28°0′8″N, 108°24′7 ″E	7
31	Wangmo	Guizhou	25°14′38″N, 106°5′59″E	7
32	Ziyun	Guizhou	25°44′43″N, 106°4′57″E	16
33	Shilin	Yunnan	24°52′6″N, 103°19′44″E	12
34	Kunming	Yunnan	24°46′2″N, 102°47′59″E	9
35	Binchuan	Yunnan	25°49′51″N, 100°34′12″E	14
36	Liuku	Yunnan	25°50′45″N, 98°51′15″E	6
37	Jinghong	Yunnan	21°57′17″N, 100°45′43″E	10
38	Jinghong	Yunnan	21°56′4″N, 100°43′13″E	8
39	Mengla	Yunnan	21°29′37″N, 101°34′14″E	6

**Table 5:** Definition and numbering of the landmarks (L.).

L. of forewing	Definition
1	Base of the radius, interaction between the radius and subcostal
2	Cross point of Anal vein and the vertex vertical line (width of wing)
3–8	Interaction between the radius branch (Rs) and the edge of the wing
9	The radius extension on front edge
10	The base of Precostal Vertex
11	The tip of Precostal Vertex
12	Cross point of Costa vein and the vertex vertical line (width of wing)
13	The base of Precostal Vertex
14	Vertex of the anal area
15	Cross point of hind edge and vertex vertical line (width of wing)
16	Interaction of the cubitus
17–19	Interaction of the radius
**L. of hindwing**	
1	Base of the subcosta
2	Base of the jugal fold
3	The radius extension on front edge
4	Vertex of the radial area
5	Interaction between the media and the edge of the wing
6	Interaction between the cubitus and the edge of the wing
7	Anal area at the edge of the central sag of the wing
8–11	Interaction between the jugal fold and the edge of the wing

Forewing with 19 landmarks and hind wing with 11 landmarks.
